# Characterization of the complete mitogenomes of the Asian Paradise Flycatcher *Terpsiphone paradisi* (Monarchidae) and phylogenetic analysis

**DOI:** 10.1080/23802359.2020.1781561

**Published:** 2020-06-22

**Authors:** Xiang Hou, Hao Yuan, Xiao-Juan Du, Hui-Sheng Gong, Gang Chang, Chao Yang, Yan Wang

**Affiliations:** a Shaanxi Institute of Zoology, Xi’an, China; b School of Life Sciences, Shaanxi Normal University, Xi’an, China

**Keywords:** Mitogenome Asian Paradise Flycatcher, *Terpsiphone paradisi*, phylogenys

## Abstract

The mitogenome of *Terpsiphone paradisi* is 16,951 bp in length and consists of 22 tRNA genes, two rRNA genes, 13 protein-coding genes and one control region. The nucleotide frequencies of As, Ts, Cs, Gs of the mitogenome is 31.0%, 24.7%, 29.8% and 14.5%, respectively. All PCGs start with typical ATN codon with the exception of COI genes, which use GTG as the initiation codon, and Most PCGs end with AGG, AGA, TAA, or TAG, except for COII, COIII and ND4, which terminated with T instead. Phylogenetic analysis indicated that genetic distances of *T. paradisi* and *Terpsiphone atrocaudata* was closer than other species.

The Asian Paradise Flycatcher *Terpsiphone paradisi* is a medium-sized wild bird species that inhabits evergreen, deciduous, and secondary growth forests throughout Asia with elevations ranging from sea level to 1,500 meters (Ngoenjun and Sitasuwan 2010). *T. paradisi* exhibits sexual dimorphism, which males having two color morphs, rufous and white, with conspicuously broad blue eye-rings and greatly elongated central pair of tail feathers, while females have only one morph, dull rufous-brown, with gray eye-rings and a short tail (Sibley and Monroe 1990; Mizuta and Yamagishi 1998a). The breeding pairs of *T. paradisi* are monogamous, with both males and females involved in nesting, hatching, brooding, and feeding of the young (Mizuta and Yamagishi 1998b; Das & Adhikari 2019). Although there have been some reports on the behavior of *T. paradisi*, the research on their molecular biology has been seriously hindered by the limitation of molecular data.

In this study, we sequenced and analyzed the mitogenome of *T. paradisi* with the GenBank accession number was MT554195. Sample of *T. paradisi* was a female adult that dead naturally during the breeding season, and was collected from Lantian County, Xi’an (34°21′ N, 109°50′ E). The specimens (proof number: SD01) were deposited in the animal specimens museumof Shaanxi Institute of Zoology, Shaanxi province, China.

Genomic DNA was prepared with a paired-end (2 × 150) libraries followed by next-generation sequencing on the Illumina Hi-Seq Xten platform, and 7,227,213 paired-end raw reads were obtained. After removing regions with a Phred score of <10, we yield the clean reads. Then MITObim version 1.9 (Hahn et al. 2013) was used to assemble the clean reads with the complete mitogenome of *T. atrocaudata* (GenBank: KT901458) as a reference. A total of 174,261 individual mitochondrial reads were mapped to the reference mitogenome, gave an average coverage of 282.8X. Geneious version 10.1.2 and MITOchondrial genome annotation Server (MITOS; http://mitos.bioinf.uni-leipzig.de/index.py) was used for annotation of protein coding genes in the mitogenome and manually inspected to predict transfer RNA (tRNA) and ribosomal RNA (rRNA) genes.

The complete mitogenome of *T. paradisi* is 16,951 bp in length, consisting of 22 tRNA genes, two rRNA genes, 13 protein-coding genes (PCGs) and one control region. The nucleotide frequencies of *T. paradisi* mitogenome are as follows: A = 31.0%, T = 24.7%, C = 29.8%, G = 14.5%, and A + T = 55.7%. The length of the 22 tRNA genes ranked from 64 bp to 75 bp, and all the tRNA genes have the typical cloverleaf secondary structures, except for *tRNA-Ser(AGY)* which the DHU arm has been replaced by a simple loop. The two rRNA genes located between *tRNA-Phe* and *tRNA-Leu*, separated by *tRNA-Val,* are 969 bp in srRNA and 1596 bp in lrRNA. All PCGs begin with the typical ATN start codon with the exception of COI genes, which use GTG as the initiation codon. And most PCGs end with AGG, AGA, TAA, or TAG, while COII, COIII and ND4 have the incomplete stop codon T. The control region, which is located between *tRNA-Glu* and *tRNA-Phe*, is 1350 bp in length and has 59.0% AT content.

To investigate the phylogenetic positions of *T. paradisi*, a phylogenetic analysis was built based on 13 PCGs and two rRNA genes with 21 ingroups and one outgroup (Figure 1). Firstly, we used MEGA7.0 (Kumar et al. 2016) to alignment DNA sequences individually and used SequenceMatrix v1.8 (Vaidya et al. 2011) to connect the alignment of individual genes. Then, PartitionFinder v2.1.1 (Lanfear et al. 2012) was used to determine the optional model and partition scheme of evolution. Finally, RaxML v8.2.12 (Stamatakis 2014) was used to built the Maximum likelihood tree with 1000 bootstrap replicates. The phylogenetic analysis showed that *T. paradisi* clustered together with *T. atrocaudata* with high bootstrap support, which was consistent with the morphological and molecular evidence, indicating that *T. paradisi* has similarities with *T. atrocaudata.* Unfortunately, due to lake of sequence dates, the topological structure of *Terpsiphone* should be futher investigated (Bristol et al. 2013).

**Figure 1.**
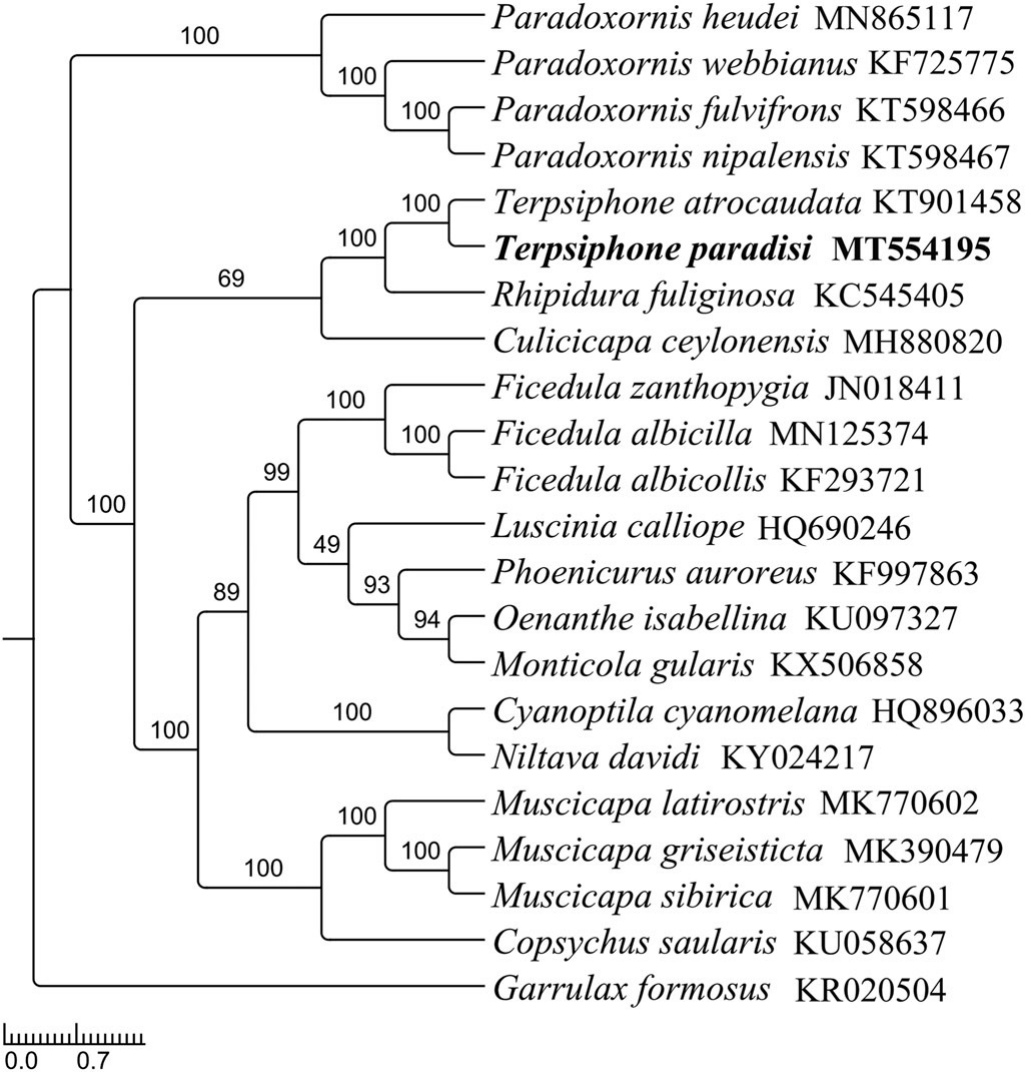
The maximum likelihood tree of *T. paradisi* and other 21 species including 20 close-related species and one outgroup species based on mitochondrial PCGs and rRNAs concatenated data. All species were downloaded in GenBank, and the accession number was given with species names. (Numbers on nodes are bootstrap values).

## Data Availability

The data that support the findings of this study are openly available in NCBI at https://www.ncbi.nlm.nih.gov/, reference number [MT554195], or available from the corresponding author.
